# Book Reviews

**Published:** 1916-06

**Authors:** 


### BOOK REVIEWS

1915 COLLECTED PAPERS OF THE MAYO CLINIC, Rochester, Minn. Octavo of 983 pages, 286 illustrations. Philadelphia and London: W. B. Saunders Company, 1916. Cloth, $6.00 net; Half Morocco, $7.50 net.

This 1915 edition is the seventh volume of the papers of the Mayo Clinic and comes to the public in response to the demand the sales and distribution of its predecessors have made.

The papers are prepared by members of the Clinic at Rochester from material there presented. They have been read either at the hospital or elsewhere before other medical societies and they are assembled here to put in permanent form some of the results accruing to the work of the famous Clinic. The wealth of material is so great that some of the papers prepared have been abstracted rather than completely published. Those that have serial relation to a subject that is carried on from year to year are all published in full, while in addition there are monographs upon such topics as seem worthy. The size of the book, 983 pages, gives an idea of the quantity of reading offered and the source speaks finally for the quality. It would be invidious to dwell upon any small number of the essays in critical review because every one is worthy of careful reading. Moreover, the world now knows the work of the Mayo Clinic and needs only to be told that the Collected Papers are out and ready for distribution. We shall carry no coals to Newcastle but just make the announcement that for all those who are desirous of having the book, it is at hand in all its excellence.

---

GYNECOLOGY. By William P. Graves, M.D., F.A.C.S., Professor of Gynecology at Harvard Medical School. Octavo volume of 770 pages with 424 original illustrations, 66 of them in colors. Philadelphia and London: W. B. Saunders Company, 1916. Cloth, $7.00 net; Half Morocco, $8.50 net.

Here is a book for every student in Gynecology. Not only should it make an excellent textbook for the undergraduate, but for the teacher and surgeon it will make an invaluable assistant. The great field of its research has been opened wide to the reader and much that has been obscure is made plain by the skill in delineation of the writer. A man who can make his own halftones as Graves does in almost every instance throughout the book, sees things and is able to tell of them more clearly and accurately than the man who has not the picture eye. Oftentimes in a description by a writer lacking in technique, the connecting line or the supporting arch is omitted or obscured and the student wonders what it is all about or how the thing can possibly have happened as it seems to present itself. Not so here. The pictures whether of pen or brush are complete and the discussions are lucid and reliable.

The arrangement of the subject matter is good and convenient. The theory is discussed and the pathology indicated by large microscopic plates with full legends under them. They were made for this book and have not been taken from other sources where the text described them. Then when the reader has absorbed the theory, he easily finds the practice in another section where once more cuts and legends are so plain that he who runs may read.

One of the valuable sections is upon "Technic." It takes the student along correctly from the time the patient first comes to his attention until the last things have been done in the way of care and tells him how to handle himself. It teaches him to avoid many natural errors and gives him for the reading, the advantages of the cumulative experience of the writer's work. The book should be the standard for textbooks in the medical colleges.

---

SOCIAL TRAVESTIES AND WHAT THEY COST. By D. T. Atkinson, M.D. 12mo, 152 pages, $1.00. New York: Vail-Ballou Co.

This small volume presents again in rather more courageous form the deplorable results of failure to educate the child in matters of the physiology of the sexual and procreative relations.

The writer speaks as a doctor who is honestly trying to make things better and who never has a thought that is sensational in character. He lays the blame where it belongs and sheds no little light upon the subject, but he misses or fears to say what the remedy for the miserable mess is.

That is a severe criticism perhaps, but no unkindness is intended. We have been so long dodging the issue and avoiding seeing what we are looking at in this matter that we have come to rely falsely upon supports that have nothing to do with it, and from these bases we continue to attempt to wield an influence that time has shown to be of no avail. Legislation against the results of sexual propinquity is as absurd as a Kansas legislature would be in declaring hurricanes illegal, and the giving of the franchise to women, which the author recommends as a probable remedy, has about the same value.

He speaks of some "inherent" and "instinctive" qualities which are really acquired or elemental and one may be misled by such concepts to avoid placing the blame upon education, where it belongs. However, that is not his intention, and the qualities he notes are worthy of the attention of the most skilled advisors.

There are some statements open to question, but here again, the use of words may be the trouble. For example he says that "'this institution (prostitution) has never existed among barbarous peoples." Harlotry has been the theme of the most ancient writers generally read. Witness David's lamentations over his manifest and hidden syphilides. They excite ridicule rather than pity because of their childish and rebellious qualities as well as for their reliance upon exorcisms for cure. Farther back when the relabeling of wives as sisters was the fashion and the marrying of half-sisters was in vogue—customs that could escape being called barbarous only by making them something worse—the mournful and expiatory conduct of Abimelech and a Pharaoh speak for the presence of prostitution before Lot stumbled away from Sodom. The blame cannot be laid upon Western civilization especially any more than the diseases damning the people at present can be traced to their source.

The book tells of many evils and hypocrisies and bravely shows the remedy for most of them. It will make any man think and it is worth while. If it has left something unsaid, the silence may be due to the undesirability at this time of exciting antagonisms which would of themselves obscure the struggle; a future study may present them to view.

---

THE MORTALITY FROM CANCER THROUGHOUT THE WORLD. By Frederick L. Hoffman, LL.D., F.S.S., F.A.S.A., Statistician The Prudential Insurance Company of America; Chairman Committee on Statistics, American Society for Control of Cancer; Member American Association for Cancer Research; Associate Fellow American Medical Association; Associate Member American Academy of Medicine. 826 Pages and Numerous Tables. Cloth. The Prudential Press, Newark, N. J.

This is a remarkable book in many ways. First, it is presented to the profession by a great Life Insurance company solely for the furtherance of the common interests the company, the profession and the people have together in the death rate of 80,000 annually from malignant disease, and, second, because the work has been done by a statistician who knows something more than figures and whose work and investigations have received the high approval of the leading medical associations of the world.

It tells in statistics the story of the development of cancer as a world problem among the thousands of cases coming annually to the attention of the life insurance companies. It draws conclusions from them and presents ever so many views that the purely medical student would not see or notice. The power of research and of follow-up that none but a large company could have has been used most interestingly and in the text the whole story is told convincingly. Later on the book has numerous tables and cross records where one may revel in figures if he is so minded and find that there is a general tendency toward a change in the human race uncertain conditions toward cancer. That would be frightful, for already the disease is the third largest in fatality among men of 45 or over, but the remedy is suggested. The whole study is in close co-operation with Cancer research societies and laboratories in this country and it is here by the way, that the best work is being done.

The thanks of the Profession are due The Prudential Company and Dr. Hoffman for this excellent book.

---

INTERNATIONAL CLINICS, Vol. 2, Twenty-Sixth Series. A Quarterly of Illustrated Clinical Lectures and Especially Prepared Original Articles on All the Divisions of Medicine and Surgery, by Leading Members of the Medical Profession Throughout the World. Edited by H. R. M. Landis, M.D., with the Collaboration of Many Well Known Men. Philadelphia and London: J. B. Lippincott Company.

This is the latest of this well-known series of books and its contents show the care and thoughtfulness of its editor in selecting from a wealth of material fine essays upon topics in the progress in medical science. It would be impossible to review the book without considering carefully each of the essays and even excerpting most of them. The reputation the Series has already earned is fully sustained. There are four Articles under Treatment, seven under Medicine, two under Psychiatry, two under Obstetrics, three under Public Health and six under Surgery. Every one of them is timely and altogether they give the reader the satisfaction of feeling that he is up-to-date in the subjects of which they treat.

The book will be welcome to subscribers and alluring to those who have the Series under advisement.

LATERAL CURVATURE OF THE SPINE and Round Shoulders. By Robert W. Lovett, M.D., Professor of Orthopedic Surgery, Harvard Medical School; Surgeon to the Children's Hospital, Boston; Surgeon in Chief to the Massachusetts Hospital School, Canton; Consulting Orthopedic Surgeon to the Boston Dispensary; Member American Orthopedic Association, and of several Foreign Societies. Third Edition, with 180 illustrations and 213 Pages. Cloth, $1.75 net. Philadelphia: P. Blaikiston's Sons & Co.

Here Scoliosis receives the most profound attention and the results of a keen and skilled observer's study and experience are placed before the rader lucidly and attractively. The history of the malady has been taken up advisedly and the various methods of treatment have been analyzed. Notably that of forcible correction has been considered and been brought to date in this third edition.

X-Ray work is studied and classified so far as it can be and the conclusions it warrants have been drawn.

The effect of the displacements of the spine upon the viscera is shown and commented upon and this of itself makes the book of value to the general practitioner who may be in the habit of thinking of scoliosis as a trouble exclusively for the orthopedist to consider.

As a monograph for special workers it would seem to be invaluable and it is commended as such.

---

HOSPITAL LABORATORY METHODS for Students and Clinicians. By Frank A. McJunkin, A.M., M.D., Professor of Pathology Marquett University School of Medicine, Milwaukee, Wis. One Colored Plate and 93 Illustrations. 139 Pages. Cloth, $1.25 net. Philadelphia: P. Blaikiston's Son & Co.

This handy little book is filled with valuable things. The reader is quickly told or reminded of the best way to do the tests daily practice requires, and the other ways are left out. The details are excellent and the instructions to be given to patients as to their part in the collecting of specimens or the co-operation they are expected to give at other times are valuable. To the student and worker, the book will be of assistance on the laboratory bench, while to the man who has his work done for him, it will give timely advice as how best to get specimens and smears.

### BOOKS RECEIVED.

Sutton: Diseases of the Skin.. The C. V. Mosby Co.

Austin: Diseases of the Gastrointestinal Canal and their Treatment. The C. V. Mosby Co.

Hazen: Skin Cancer. The C. V. Mosby Co.

